# Elevated serum cardiotrophin-1 and interleukin-17 levels in psoriasis

**DOI:** 10.1007/s11845-026-04373-4

**Published:** 2026-04-14

**Authors:** Fatma Sengul-Bag, Fikret Akyurek, Halil Guven, Fatma Tuncez-Akyurek

**Affiliations:** 1 Department of Biochemistry, Faculty of Pharmacy, University of Adıyaman, Adıyaman, Türkiye; 2 Department of Medical Biochemistry, Faculty of Medicine, University of Selçuk, Konya, Türkiye; 3 Department of Dermatology, Faculty of Medicine, University of Selçuk, Konya, Türkiye

**Keywords:** Cardiometabolic risk, Cardiotrophin-1, Dyslipidemia, Interleukin-17, Psoriasis

## Abstract

**Background:**

Psoriasis is a systemic, immune-mediated chronic disease in which the interleukin-23 (IL-23)/T-helper 17 (Th17) axis and its key effector cytokine, IL-17, play a central role. Cardiotrophin-1 (CT-1), a multifunctional cytokine from the IL-6 family, is involved in cardiovascular and metabolic homeostasis. This study aimed to evaluate serum levels of CT-1 and IL-17 in psoriasis, investigating their relationship with clinical parameters and their potential role as biomarkers for systemic inflammation and increased cardiometabolic risk.

**Methods:**

This study included 64 patients diagnosed with psoriasis vulgaris and 64 age- and sex-matched healthy controls. Serum CT-1 and IL-17 levels were measured using enzyme-linked immunosorbent assay (ELISA). Disease severity was assessed using the Psoriasis Area and Severity Index (PASI) and the Dermatology Life Quality Index (DLQI).

**Results:**

Serum levels of both CT-1 and IL-17 were significantly higher in the psoriasis group compared to the control group (p < 0.001 for each). A strong positive correlation was found between the two cytokines (ρ = 0.890, p < 0.001). CT-1 showed stronger correlations with lipid parameters than IL-17. However, no significant correlation was observed for either cytokine with PASI or DLQI. Receiver operating characteristic (ROC) analysis demonstrated that CT-1 had stronger diagnostic accuracy (AUC: 0.915, 95% CI:0.869–0.961) for distinguishing psoriasis patients from healthy controls compared to IL-17 (AUC: 0.733, 95% CI:0.645–0.822).

**Conclusions:**

This study shows that CT-1 and IL-17 levels are significantly elevated in psoriasis and suggests that this increase may be associated with systemic inflammation and dyslipidemia. The observed discriminative performance of CT-1 supports its potential relevance as a marker of systemic inflammatory and cardiometabolic involvement, although its clinical utility requires further clarification.

## Introduction

Psoriasis is a systemic, immune-mediated chronic disease driven by inflammatory processes and characterized by skin and joint involvement. Affecting approximately 2–3% of the population, it is considered a significant public health problem [1]. The pathogenesis of the disease is highly complex, involving an interplay of genetic predisposition, environmental triggers, and immune system dysregulation [2]. The interleukin-23 (IL-23)/T-helper 17 (Th17) axis and its key effector cytokine, IL-17, are central to psoriatic inflammation. IL-17 promotes keratinocyte proliferation and triggers the release of other pro-inflammatory cytokines (e.g., IL-6, tumor necrosis factor-alpha [TNF-α]), thereby sustaining disease pathogenesis [3–5]. IL-17 levels are significantly elevated in psoriatic skin lesions, and this increase is closely associated with disease severity. Furthermore, the reduction in clinical Psoriasis Area and Severity Index (PASI) scores following treatment with IL-17 inhibitor agents confirms the central role of this cytokine in disease pathogenesis [6, 7].

Psoriasis is not confined to skin lesions but is also associated with systemic inflammation and metabolic disorders [8, 9]. The literature reports that cardiometabolic risk factors (such as hyperlipidemia, hypertension, type 2 diabetes, and coronary artery disease) are more prevalent in individuals with psoriasis than in healthy individuals [10, 11]. Specifically, as psoriasis severity increases, the prevalence of diabetes and cardiovascular disease also rises significantly [11]. It has been demonstrated that psoriasis can independently increase the risk of myocardial infarction, stroke, and cardiovascular mortality [1, 10]. These data indicate that psoriasis is not merely a dermatological disorder but a systemic inflammatory syndrome [8, 12].

Cardiotrophin-1 (CT-1), a multifunctional cytokine belonging to the IL-6 family, exhibits both protective and inflammatory effects, particularly in the cardiovascular system [13]. Produced by the heart, vascular endothelium, smooth muscle cells, macrophages, and adipocytes, CT-1 is released in response to stimuli such as hypoxia, mechanical stress, angiotensin II, and oxidative stress. By signaling through the glycoprotein 130 (gp130)/leukemia inhibitory factor receptor beta (LIFRβ) receptor complex and activating the phosphoinositide 3-kinase (PI3K)/protein kinase B (Akt), Janus kinase (JAK)/signal transducer and activator of transcription (STAT), and nuclear factor kappa B (NF-κB) pathways, CT-1 regulates cellular proliferation, anti-apoptotic responses, and the production of inflammatory mediators [13, 14]. It has been reported to contribute to atherosclerotic processes, including endothelial dysfunction, foam cell formation, and smooth muscle cell migration, thereby promoting pathological vascular remodeling [13, 15]. Furthermore, CT-1 levels are elevated in inflammation-associated conditions such as hypertension, left ventricular hypertrophy, heart failure, metabolic syndrome, and obesity, suggesting it may serve as both a marker and a mediator of tissue damage [13, 16, 17].

Given the widespread presence of systemic inflammation and increased cardiometabolic risk observed in individuals with psoriasis, CT-1 is thought to be both a consequence of inflammation and a potential biomarker for associated cardiovascular comorbidities. Its sharing of common signaling pathways with IL-17 and IL-6 suggests that CT-1 may be an important mediator in the systemic manifestations of psoriatic inflammation. Although IL-17 has been extensively studied in psoriasis, the potential involvement of CT-1 in this disease has been only sparsely investigated. In particular, its relationship with systemic inflammation and cardiometabolic risk in psoriasis remains unclear. Therefore, this study aimed to evaluate IL-17 and CT-1 levels in patients with psoriasis, to investigate the relationship between these two biomarkers, and to explore the potential relevance of CT-1 in the context of systemic inflammation and cardiometabolic risk.

## Materials and methods

### Study population

This study included 64 patients with psoriasis vulgaris who were evaluated in the Department of Dermatology, Selçuk University Faculty of Medicine, and 64 age- and sex-matched healthy controls. Patients were enrolled from individuals presenting to the outpatient clinic who were either newly diagnosed or in remission and had not received systemic or biological treatment within the preceding three months. The control group consisted of individuals with no dermatological, autoimmune, or chronic systemic diseases, as confirmed by a general health examination. Individuals aged 18–65 years who had not received systemic or biological agent therapy in the preceding three months and had used only topical treatment in the last month were included. Exclusion criteria were a diagnosis of psoriatic arthritis or any other autoimmune or inflammatory disease; a history of type 1 or type 2 diabetes, hypertension, hyperlipidemia, coronary artery disease, myocardial infarction, heart failure, or arrhythmia; hepatic or renal failure; active smoking or alcohol use; pregnancy or lactation; a history of infection or use of antibiotics or anti-inflammatory drugs within the last month; and a history of immunosuppressive or biological therapy within the preceding three months.

The study protocol was approved by the Selçuk University Faculty of Medicine Local Ethics Committee (Decision No: 2025/223) and conducted in accordance with the principles of the Declaration of Helsinki.

### Clinical assessments

The PASI was used to assess psoriasis severity. PASI is a semi-quantitative scoring system that considers both the extent of body surface area involvement and the severity of clinical features (erythema, infiltration, and desquamation) of psoriatic lesions. For assessment, the body was divided into four anatomical regions: head, trunk, upper extremities, and lower extremities. The involved area in each region was scored from 1 to 6 based on the percentage of surface coverage. The severity of erythema, infiltration, and desquamation in each region was graded from 0 (none) to 4 (severe). The sum of these three severity scores was multiplied by the area score for the respective region and then by a weighting factor reflecting the region’s contribution to total body surface area (head: 0.1; upper extremities: 0.2; trunk: 0.3; lower extremities: 0.4) to yield the regional score. The sum of the scores from all four regions gave the total PASI score [18]. Additionally, quality of life was assessed using the Dermatology Life Quality Index (DLQI), and nail involvement was recorded based on clinical examination findings.

### Biochemical measurements

Serum levels of albumin, glucose, triglycerides, total cholesterol, high-density lipoprotein cholesterol (HDL-C), low-density lipoprotein cholesterol (LDL-C), aspartate aminotransferase (AST), and alanine aminotransferase (ALT) were measured using a photometric method on a Roche Cobas c702 analyzer (Roche Diagnostics, Mannheim, Germany). Non-HDL cholesterol was calculated using the formula [total cholesterol − HDL-C]. The estimated glomerular filtration rate (eGFR) was determined using the Chronic Kidney Disease Epidemiology Collaboration (CKD-EPI) formula.

Serum levels of CT-1 and IL-17 were determined using a Human CT-1 ELISA Kit (Cat. No: E1228Hu, BT LAB Bioassay Technology Laboratory, Zhejiang, China) and a Human IL-17 ELISA Kit (Cat. No: E0142Hu, BT LAB Bioassay Technology Laboratory, Zhejiang, China), respectively, following the manufacturers’ instructions. Measurements were based on the sandwich-type enzyme-linked immunosorbent assay (ELISA) principle. The measurement ranges for CT-1 and IL-17 were 3–900 ng/mL and 2–600 ng/L, with sensitivity limits of 1.65 ng/mL and 1.06 ng/L, respectively. All reagents were brought to room temperature before the assays, and standard curves were prepared according to the manufacturer’s protocol. Absorbance was read at 450 nm using a CLARIOstar microplate reader (BMG LABTECH, Ortenberg, Germany). Analytical precision was assessed based on the reproducibility criteria reported by the manufacturer, with intra-assay coefficients of variation (< 8%) and inter-assay coefficients of variation (< 10%) being maintained. Results were calculated from the standard curve absorbance values, and serum concentrations were expressed in ng/mL or ng/L. Serum samples were processed within 30 min of collection, aliquoted, and stored at − 80 °C until measurement. All samples were analyzed within 6 months of storage and underwent a single freeze–thaw cycle.

### Statistical analysis

All statistical analyses were conducted with R statistical software, version 4.5.0 (R Foundation for Statistical Computing, Vienna, Austria). Normality of data distribution was evaluated using the Shapiro–Wilk test alongside visual inspection of Q-Q plots. The homogeneity of variances was assessed via Levene’s test. For comparisons between the two groups, the Student’s t-test was applied to normally distributed data, whereas the Mann–Whitney U test was used for data that deviated from normality. Categorical variables were compared using either the Chi-square test (χ2) or Yates’ correction for continuity, depending on the sample size. Continuous data are summarized as mean ± standard deviation or as median with interquartile range (1st quartile–3rd quartile). Categorical data are expressed as frequencies and percentages [n (%)]. Spearman’s rank correlation coefficient (ρ) was employed to examine associations between serum levels of CT-1 and IL-17 and various clinical and biochemical parameters in psoriasis patients. The diagnostic potential of CT-1 and IL-17 to differentiate psoriasis patients from healthy controls was evaluated using receiver operating characteristic (ROC) curve analysis. Key performance metrics including the area under the curve (AUC), sensitivity, specificity, positive predictive value (PPV), and negative predictive value (NPV) were derived. The optimal cut-off point was identified by maximizing Youden’s index (*J* = Sensitivity + Specificity − 1). A multiple linear regression analysis was performed to identify the independent predictors of serum CT-1 levels, adjusting for potential confounding factors including disease status, age, sex, and lipid parameters. Statistical significance was defined as a two-tailed p-value of less than 0.05. In addition to p values, effect size measures were calculated to quantify the magnitude of between-group differences. Rank-biserial correlation (r) was reported for Mann–Whitney U tests, Cohen’s d for independent-samples t tests, and Cramér’s V for categorical comparisons.

## Results

**Fig. 1:**
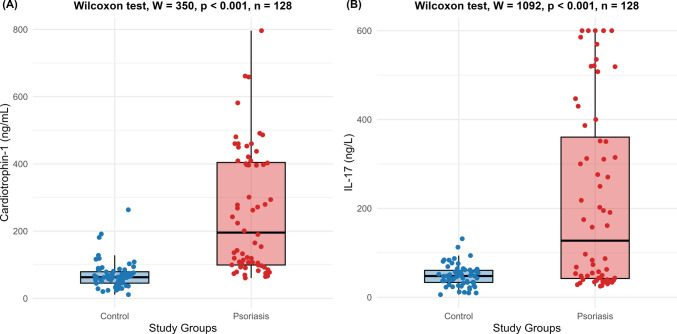
The serum level of (A) CT-1 (ng/mL) and (B) IL-17 (ng/L) in psoriasis patients and healthy controls. The black line inside the box represents the median and interquartile range (IQR), and blue and red dots represent individual values in the control and psoriasis groups, respectively. Wilcoxon test: Un-paired Mann–Whitney U test. IL-17, interleukin-17

The study groups were similar in terms of age and sex distribution (p = 0.943 and p = 0.595, respectively). Serum albumin, eGFR, glucose, triglycerides, total cholesterol, LDL-C, HDL-C, and non-HDL cholesterol levels showed statistically significant differences between psoriasis patients and healthy controls, whereas ALT and AST levels did not differ significantly. Psoriasis patients had significantly higher serum levels of CT-1 (ng/mL) and IL-17 (ng/L) compared to controls (both p < 0.001). Clinical evaluation revealed that 32.8% of psoriasis patients had nail involvement. The median disease duration was 60 (35.25–123) months, with a median PASI score of 2.65 (0.8–4) and DLQI of 2 (0–5). These results are presented in detail in Table 1 and illustrated in Fig. 1.

**Table 1:** Demographic characteristics, clinical parameters, and serum biochemical measurements in psoriasis patients and healthy controls

Variable	Control (n = 64)	Psoriasis (n = 64)	p value	Effect size
Demographic characteristics				
Age (years)	38 (27.75–50)	37 (26.75–50)	0.943^1^	0.006
Sex (Female/Male)	30 (46.9%)/34 (53.1%)	34 (53.1%)/30 (46.9%)	0.595^2^	0.047
Laboratory parameters				
Albumin (g/dL)	4.8 (4.6–4.9)	4.5 (4.38–4.6)	< 0.001^1^	0.536
eGFR (mL/min/1.73m^2^)	119.91 ± 14.34	111.57 ± 17.47	0.004^3^	0.522
Glucose (mg/dL)	86 (83.75–93.25)	92 (86.5–102)	< 0.001^1^	0.317
Triglycerides (mg/dL)	77 (65–90.5)	161.5 (123.25–206.5)	< 0.001^1^	0.780
Total cholesterol (mg/dL)	141.5 (124.5–157.5)	188.5 (178.75–205)	< 0.001^1^	0.764
LDL-C (mg/dL)	88.18 ± 22.28	114.3 ± 29.75	< 0.001^3^	0.994
HDL-C (mg/dL)	51.5 (45.75–57)	41 (37–48)	< 0.001^1^	0.413
Non-HDL cholesterol (mg/dL)	88.8 ± 23.8	147.2 ± 28.0	< 0.001^3^	2.250
ALT (IU/L)	15.5 (12–21)	17.5 (13–33)	0.125^1^	0.136
AST (IU/L)	16 (14–20)	17 (14.75–22.25)	0.115^1^	0.139
CT-1 (ng/mL)	62.94 (45.35–79.23)	195.67 (99.35–404.33)	< 0.001^1^	0.715
IL-17 (ng/L)	47.75 (33.35–60.57)	127.37 (42.23–360.43)	< 0.001^1^	0.403
Clinical characteristics				
Nail involvement (yes/no)	NA	21 (32.8%)/43 (67.2%)		
Disease duration (months)	NA	60 (35.25–123)		
PASI	NA	2.65 (0.8–4)		
DLQI	NA	2 (0–5)		

^1^Mann–Whitney U test, ^2^ Chi-square test with Yates continuity correction, ^3^ Independent Samples T-Test. Categorical variables are presented as numbers and percentages [n (%)], while continuous variables are expressed as mean ± standard deviation, median with interquartile range (1st quartile–3rd quartile), or count (n) and percentage (%), as appropriate. A p-value < 0.05 was considered statistically significant. Effect size is presented as rank-biserial correlation (r) for Mann–Whitney U tests, Cohen’s d for independent-samples t-tests, and Cramér’s V for categorical comparisons. CT-1, cardiotrophin-1; IL-17,interleukin-17; LDL-C, low-density lipoprotein cholesterol; HDL-C, high-density lipoprotein cholesterol; Non-HDL cholesterol, total cholesterol minus HDL-C; ALT, alanine aminotransferase; AST, aspartate aminotransferase; eGFR, estimated glomerular filtration rate; PASI, Psoriasis Area and Severity Index; DLQI, Dermatology Life Quality Index; NA, not applicable

Spearman’s *rho* correlation analysis revealed a very strong positive correlation between serum CT-1 and IL-17 levels (ρ = 0.890, p < 0.001) in patients with psoriasis. A comparative analysis of the correlation patterns showed that CT-1 levels demonstrated significant positive correlations with triglycerides (ρ = 0.529, p < 0.001), total cholesterol (ρ = 0.604, p < 0.001), LDL-C (ρ = 0.345, p < 0.001), and non-HDL cholesterol levels (ρ = 0.587, p < 0.001), alongside significant negative correlations with albumin (ρ = −0.329, p < 0.001) and HDL-C (ρ = −0.200, p = 0.023). Similarly, IL-17 levels were positively correlated with triglycerides (ρ = 0.259, p = 0.003), total cholesterol (ρ = 0.295, p < 0.001), LDL-C (ρ = 0.234, p = 0.008), and non-HDL cholesterol (ρ = 0.282, p = 0.001), and negatively correlated with albumin (ρ = −0.179, p = 0.043). Conversely, no significant correlations were observed for either cytokine with disease activity scores (PASI, DLQI), disease duration, glucose, eGFR, or liver enzymes (ALT, AST) (Table 2).

**Table 2:** Spearman’s *rho* correlation coefficients laboratory parameters in Psoriasis patients, by CT-1 and IL-17

	CT-1		IL-17	
	Spearman’s *rho*	*p*-value	Spearman’s *rho*	*p*-value
PASI	−0.054	0.671	−0.087	0.492
DLQI	0.042	0.744	0.047	0.711
Disease duration (months)	−0.050	0.696	−0.093	0.464
Albumin (g/dL)	−0.329	< 0.001	−0.179	0.043
eGFR (mL/min/1.73m2)	−0.132	0.137	0.035	0.695
Glucose (mg/dL)	0.134	0.133	−0.011	0.906
Triglycerides (mg/dL)	0.529	< 0.001	0.259	0.003
Total cholesterol (mg/dL)	0.604	< 0.001	0.295	< 0.001
LDL-C (mg/dL)	0.345	< 0.001	0.234	0.008
HDL-C (mg/dL)	−0.200	0.023	−0.092	0.303
Non-HDL cholesterol (mg/dL)	0.587	< 0.001	0.282	0.001
ALT (IU/L)	0.108	0.227	0.043	0.632
AST (IU/L)	0.131	0.141	0.082	0.356
IL-17	0.890	< 0.001	-	-

Significant relationships denoted as bold. CT-1, cardiotrophin-1; IL-17, interleukin-17; PASI, Psoriasis Area and Severity Index; DLQI, Dermatology Life Quality Index; eGFR, estimated glomerular filtration rate; LDL-C, low-density lipoprotein cholesterol; HDL-C, high-density lipoprotein cholesterol; Non-HDL cholesterol, non–high-density lipoprotein cholesterol; ALT, alanine aminotransferase; AST, aspartate aminotransferase

According to the multiple linear regression analysis performed to evaluate whether the association between CT-1 and psoriasis was independent of lipid parameters, CT-1 was included as the dependent variable, and psoriasis status, triglycerides, LDL-C, HDL-C, age, and sex were entered as independent variables. After adjustment, psoriasis remained significantly associated with CT-1 levels (β = 200.9, p < 0.001), whereas triglycerides (p = 0.417), LDL-C (p = 0.264), and HDL-C (p = 0.089) were not significantly associated with CT-1.

**Fig. 2:**
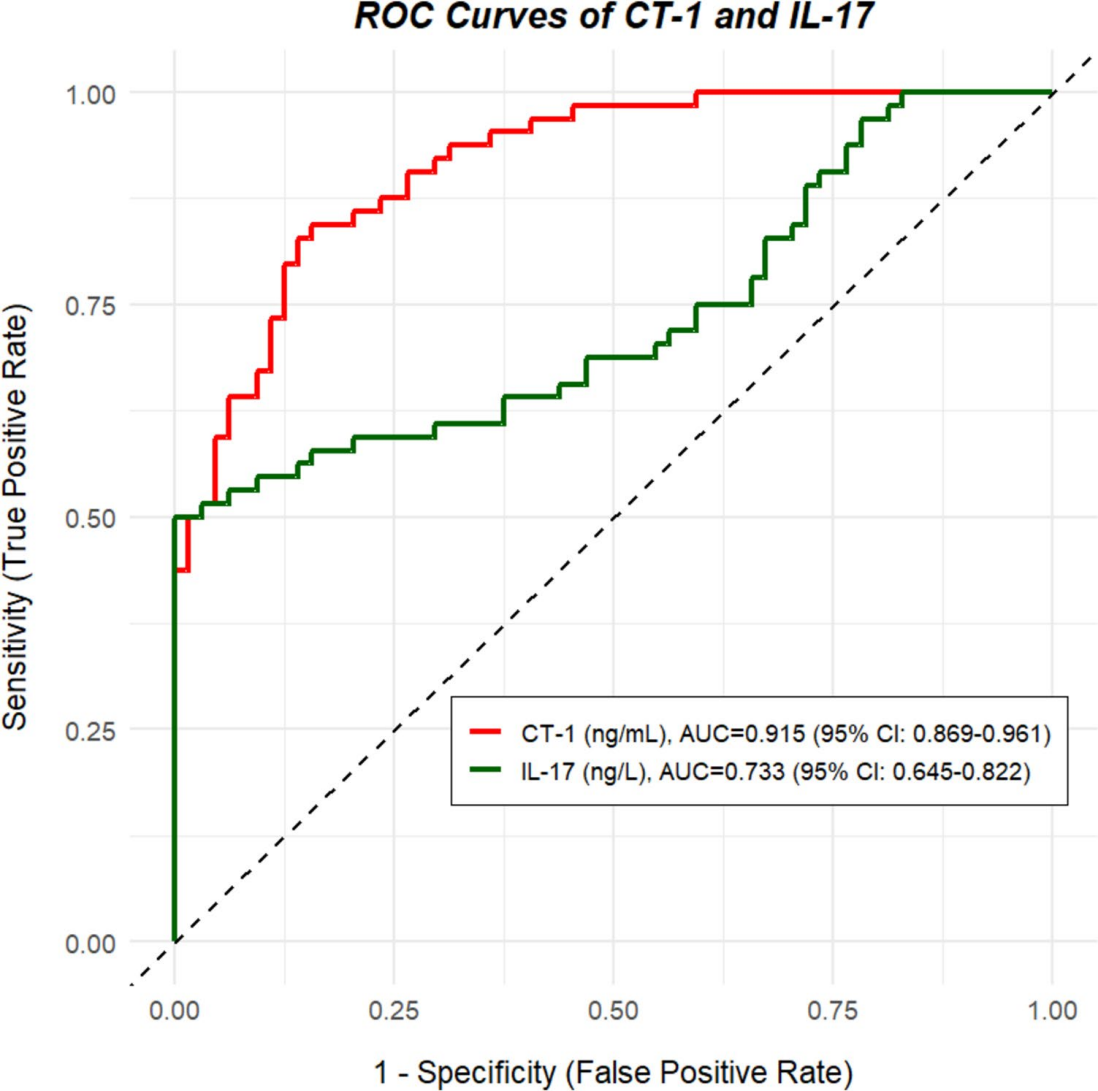
ROC curves of CT-1 and IL-17 for distinguishing psoriasis patients from healthy controls. IL-17, interleukin-17; CT-1, cardiotrophin-1; AUC, area under the curve; 95% CI, 95% confidence interval; ROC, receiver operating characteristic

ROC curve analysis was conducted to evaluate the diagnostic performance of CT-1 and IL-17 in distinguishing psoriasis patients from healthy controls. The analysis demonstrated that CT-1 had an excellent discriminative capacity, with an AUC of 0.915 (95% CI, 0.869–0.961; p < 0.001). The optimal cut-off value of CT-1 was determined as ≥ 90.2 ng/mL, which yielded a sensitivity of 84.4%, a specificity of 84.4%, a PPV of 84.4%, and an NPV of 84.4%. In comparison, IL-17 showed a moderate diagnostic accuracy, with an AUC of 0.733 (95% CI, 0.645–0.822; p < 0.001). The optimal cut-off value for IL-17 was identified as ≥ 144.7 ng/L, resulting in a sensitivity of 50%, specificity of 100%, PPV of 100%, and NPV of 66.7% (Table 3 and Fig. 2). Comparison of ROC curves using the DeLong test showed that the AUC of CT-1 was significantly higher than that of IL-17 (p < 0.001).

**Table 3:** ROC curve analysis and discriminative performance of CT-1 and IL-17 for distinguishing psoriasis patients from healthy controls

	ROC curve analysis			Statistical diagnostic measures			
	AUC (95% CI)	*p*-value	Cut-off	Sensitivity (%)	Specificity (%)	PPV (%)	NPV (%)
CT-1 (ng/mL)	0.915 (0.869–0.961)	< 0.001	≥ 90.2	84.4	84.4	84.4	84.4
IL-17 (ng/L)	0.733 (0.645–0.822)	< 0.001	≥ 144.7	50	100	100	66.7

AUC, area under the curve; 95% CI, 95% confidence interval; IL-17, interleukin-17; CT-1, cardiotrophin-1; ROC, receiver operating characteristic; PPV, positive predictive value; NPV, negative predictive value

## Discussion

The aim of this study was to compare serum CT-1 and IL-17 levels in patients with psoriasis and healthy controls, and to evaluate the relationship of these biomarkers with clinical parameters. Our findings showed that the concentrations of both CT-1 and IL-17 were statistically significantly higher in the psoriasis group than in the control group. Furthermore, a strong positive correlation was observed between the two parameters. These findings suggest that CT-1 may interact with the IL-17-mediated pathogenic axis in the context of the systemic inflammatory response seen in psoriasis.

The increase in serum CT-1 levels indicates that this cytokine can be activated not only under cardiac or metabolic stress but also as part of a chronic systemic inflammatory response. CT-1 concentrations are known to be markedly elevated in conditions such as ischemic heart disease, acute myocardial infarction, atherosclerosis, and heart failure [14, 15, 19–21]. Increased secretion from cardiac tissue, the primary source of circulating CT-1, following ischemic injury supports the molecule’s active role in the cardiomyocyte stress response [13]. Additionally, the elevation of CT-1 in individuals with metabolic syndrome suggests that this cytokine may serve as a link between inflammation, energy metabolism, and vascular remodeling. Collectively, these findings support the view that the CT-1 increase detected in individuals with psoriasis may reflect the underlying systemic inflammatory burden with cardiometabolic implications. Considering that CT-1 regulates cell proliferation, anti-apoptotic responses, and the production of inflammatory mediators via the JAK/STAT and MAPK pathways [22, 23], it can be concluded that this increase indicates that psoriatic inflammation is a systemic process associated with endothelial dysfunction and metabolic imbalances, rather than a pathology confined solely to the skin.

Elevated IL-17 levels highlight the central role of Th17 cells in the immunopathogenesis of psoriasis. This cytokine amplifies the proinflammatory response by promoting keratinocyte activation and neutrophil migration. Literature findings support this mechanism. Takahashi et al. reported significantly higher levels of IL-17, TNF-α, IL-12, IL-18, and vascular endothelial growth factor (VEGF) in 122 individuals with psoriasis compared to healthy controls, with these parameters showing a positive correlation with PASI score [24]. Similarly, Büyükkara Yılmaz et al. found that serum IL-17 levels increased in parallel with disease severity and were markedly elevated, particularly in the pustular subtype of psoriasis [25]. The role of IL-17 is not limited to the skin; Morsy et al. observed that this cytokine significantly increased in the presence of obesity and dyslipidemia, indicating its potential link with cardiometabolic disorders [26]. Furthermore, it has been reported that IL-17 creates a vicious cycle between inflammation and angiogenesis by increasing VEGF production, and that Th17 cell populations expand in obesity and metabolic syndrome [27, 28]. These findings support a mutual pathogenetic relationship between psoriatic inflammation and metabolic disorders at the biochemical level.

**Fig. 3:**
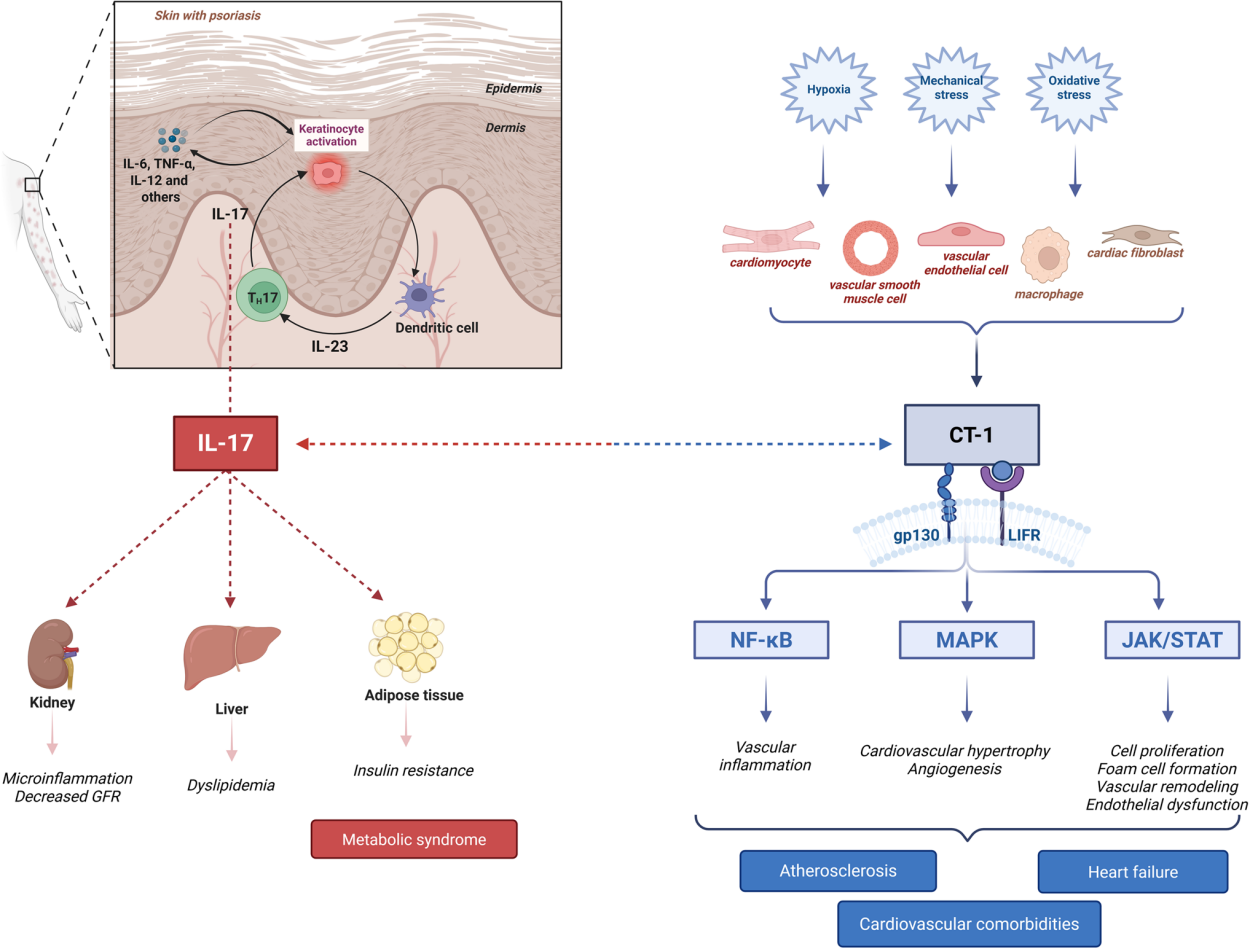
Proposed mechanistic link between IL-17–driven psoriatic inflammation and CT-1–mediated cardiometabolic dysfunction (*created with Biorender.com*). IL-17, interleukin-17; CT-1, cardiotrophin-1; IL-23, interleukin-23; TNF-α, tumor necrosis factor-alpha; NF-κB, nuclear factor kappa B; MAPK, mitogen-activated protein kinase; JAK, Janus kinase; STAT, signal transducer and activator of transcription; LIFR, leukemia inhibitory factor receptor; gp130, glycoprotein 130; GFR, glomerular filtration rate

The proinflammatory effects of IL-17 extend beyond epidermal keratinocytes to endothelial, hepatic, and renal cells, where it upregulates proinflammatory gene expression, demonstrating a systemic effect. It has been reported to act synergistically with TNF-α, IL-1β, and IL-6, activating NF-κB and MAPK signaling pathways [28]. These findings suggest that the systemic inflammation observed in psoriasis could lead to multi-organ involvement and support the idea that the serum IL-17 levels detected in our study may serve as a biomarker reflecting the systemic immune response of the disease. Moreover, the increase in IL-17 aligns with the triad of IL-9, IL-17, and VEGF reported by Midde et al.; the coordinated rise of these cytokines may establish a biochemical link between inflammation and angiogenesis [29]. IL-9 has been shown to induce IL-17 production, and this axis promotes microvascular remodeling via VEGF. IL-17 is also known to accelerate the atherosclerotic process by increasing the expression of adhesion molecules, chemokines, and VEGF in endothelial cells. This process, termed the "inflammatory march," is a key mechanism explaining how inflammation originating in the skin can disseminate via the circulation to systemic vascular structures [12]. When these mechanisms are considered together, it is plausible to propose that CT-1, through its anti-apoptotic and adaptive responses in endothelial cells, might contribute to the compensatory regulation of vascular inflammation. In this context, the positive correlation observed between CT-1 and IL-17 (Table 2) may reflect two distinct yet complementary components of the inflammatory-angiogenic process (as shown in Fig. 3).

Johansen et al. reported increased levels of IL-17A, IL-17C, and IL-17F isoforms in psoriatic lesions, along with decreased expression of certain receptor subtypes [30]. This finding highlights the tissue-specific and complex regulatory nature of IL-17 signaling. The significant correlation observed between serum IL-17 levels and CT-1 in our study suggests potential synergistic interactions between these two molecules at different stages of the inflammatory cascade. This strong correlation may reflect shared upstream inflammatory pathways, including cytokine-mediated activation of JAK/STAT and NF-κB signaling [22, 28]. Supporting this view, Schwarz et al. reported that IL-17 inhibitor therapy alters the systemic cytokine profile and leads to improvement in lipid parameters and reduced inflammatory burden [31]. The positive correlations we identified between IL-17 and triglyceride, total cholesterol, LDL-C, and non-HDL cholesterol levels further substantiate the role of this cytokine in metabolic regulation.

Buyukkara Yılmaz et al. demonstrated significantly elevated serum IL-17 levels in plaque psoriasis patients with PASI ≥ 10, particularly noting statistically significant increases in pustular subtypes [25]. This observation suggests that IL-17 may play a more determinant pathogenic role in psoriasis subtypes dominated by neutrophilic inflammation. The parallel elevation of CT-1 levels with IL-17 in our study supports the hypothesis that CT-1 may function as a regulatory factor in the vascular stress response during neutrophil-mediated inflammatory processes.

In the ROC analysis, CT-1 showed a high discriminative capacity for distinguishing psoriasis patients from healthy controls (AUC = 0.915, 95% CI: 0.869–0.961; p < 0.001). The significantly higher AUC of CT-1 compared to IL-17 further supports its stronger discriminative performance within the study sample. The optimal cut-off value of ≥ 90.2 ng/mL yielded 84.4% sensitivity and 84.4% specificity. However, because psoriasis is primarily diagnosed on clinical grounds, these findings should not be interpreted as supporting a standalone diagnostic role for CT-1. Rather, the observed discriminative performance may indicate that CT-1 reflects systemic inflammatory and cardiometabolic involvement in psoriasis. Considering that CT-1 levels are also elevated in cardiovascular pathologies such as hypertensive heart disease, coronary artery disease, and atherosclerosis [13], this molecule may represent a potential marker of systemic involvement.

In our study, CT-1 and IL-17 levels did not significantly correlate with clinical scores (PASI and DLQI). It is possible that these biomarkers act as indicators of overall systemic inflammatory burden rather than cutaneous severity. Alternatively, the absence of a correlation could simply stem from the relatively low median PASI score of our cohort, limiting our ability to observe an effect in severe disease.

The role of CT-1 in metabolic regulation includes multiple functions such as insulin resistance in adipocytes, fibrosis and remodeling in vascular smooth muscle cells, and lipid metabolism regulation in the liver [13, 22]. The negative correlation we observed between CT-1 and albumin (ρ = −0.329) aligns with the negative acute phase response, an indicator of systemic inflammation [32]. Similarly, decreased eGFR values may reflect renal effects of microinflammation and endothelial dysfunction observed in psoriasis patients. Proinflammatory cytokines such as IL-17 have been proposed to trigger immune responses on renal endothelium, potentially leading to impaired glomerular filtration [33].

The lipid profile alterations indicate a dyslipidemic pattern in patients with psoriasis. The stronger correlation of CT-1 with lipid parameters compared to IL-17 suggests that CT-1 may play a more significant pathogenic role in psoriasis-associated dyslipidemia. IL-17 has been shown to facilitate insulin resistance by increasing IL-6 expression in preadipocytes and suppressing C/EBP-α and PPAR-γ gene expression, which are crucial regulators of adipocyte differentiation [34]. This finding supports previous reports of IL-17’s association with components of metabolic syndrome [26]. Conversely, CT-1 contributes to vascular remodeling by stimulating fibrosis, proliferation, and inflammatory responses in vascular smooth muscle cells [13]. The parallel increase in CT-1 and IL-17 levels in individuals with psoriasis indicates dynamic interactions among inflammatory signaling, endothelial remodeling, and disturbances in lipid metabolism. The multivariate analysis further showed that the association between CT-1 and psoriasis persisted after adjustment for lipid parameters and demographic variables, suggesting that CT-1 elevation may not be solely related to dyslipidemia but may also reflect disease-related systemic inflammation.

This study has several limitations. The cross-sectional design precludes establishing causality in the relationships among CT-1, IL-17, and psoriasis. The limited sample size restricted detailed analyses across psoriasis subtypes. In addition, the majority of patients in our cohort had mild disease, as reflected by the low median PASI score, which may have limited our ability to detect associations between biomarker levels and disease severity. Moreover, detailed cardiometabolic parameters such as body mass index, waist circumference, and formal metabolic syndrome components were not available and therefore could not be adjusted for in the analysis, which may have influenced CT-1 levels. Single-time-point measurement of biomarker levels may not reflect their fluctuations over time. Furthermore, the single-center design may limit the generalizability of the findings. Despite these limitations, our study provides important data regarding the role of CT-1 in psoriasis and serves as a guide for future research.

In conclusion, this study demonstrates significantly elevated serum levels of CT-1 and IL-17 in patients with psoriasis, as well as a strong positive correlation between these two molecules. These findings suggest that both molecules may play interactive roles in the systemic inflammatory response of psoriasis. The high discriminative performance of CT-1 supports its potential relevance as a marker of systemic inflammation and cardiometabolic involvement in psoriasis, rather than as a primary diagnostic tool. Furthermore, CT-1's association with vascular inflammation and metabolic stress underscores its clinical importance for early detection of cardiometabolic risk in individuals with psoriasis. Future prospective studies should focus on monitoring CT-1 and IL-17 levels in patients receiving biologic therapy. The potential of these biomarkers to reflect treatment response, indicate resolution of systemic inflammation, and predict long-term cardiovascular risk warrants evaluation. Additionally, integrating CT-1 into multi-biomarker panels with conventional inflammatory markers such as CRP, TNF-α, and IL-6 could enhance the accuracy of predicting disease progression and complication risk in psoriasis.

## Data Availability

The data underlying this article will be shared on reason-able request to the corresponding author.

## References

[1] Rendon A, Schäkel K (2019) Psoriasis pathogenesis and treatment. Int J Mol Sci 20:1475. 10.3390/ijms20061475 30909615 10.3390/ijms20061475 PMC6471628

[2] Orzan OA, Tutunaru CV, Ianosi SL (2025) Understanding the intricate pathophysiology of psoriasis and related skin disorders. Int J Mol Sci 26:749. 10.3390/ijms26020749 39859462 10.3390/ijms26020749 PMC11766135

[3] Johansen C, Mose M, Ommen P et al (2015) Iκbζ is a key driver in the development of psoriasis. Proc Natl Acad Sci U S A 112:E5825-5833. 10.1073/pnas.1509971112 26460049 10.1073/pnas.1509971112 PMC4629387

[4] Singh R, Koppu S, Perche PO, Feldman SR (2021) The cytokine mediated molecular pathophysiology of psoriasis and its clinical implications. Int J Mol Sci 22:12793. 10.3390/ijms222312793 34884596 10.3390/ijms222312793 PMC8657643

[5] Wu M, Dai C, Zeng F (2023) Cellular mechanisms of psoriasis pathogenesis: a systemic review. Clin Cosmet Investig Dermatol 16:2503–2515. 10.2147/ccid.S420850 37727872 10.2147/CCID.S420850 PMC10506593

[6] Martin DA, Towne JE, Kricorian G et al (2013) The emerging role of IL-17 in the pathogenesis of psoriasis: preclinical and clinical findings. J Invest Dermatol 133:17–26. 10.1038/jid.2012.194 22673731 10.1038/jid.2012.194 PMC3568997

[7] Cua DJ, Tato CM (2010) Innate IL-17-producing cells: the sentinels of the immune system. Nat Rev Immunol 10:479–489. 10.1038/nri2800 20559326 10.1038/nri2800

[8] Campanati A, Marani A, Martina E et al (2021) Psoriasis as an immune-mediated and inflammatory systemic disease: from pathophysiology to novel therapeutic approaches. Biomedicines 9:1511. 10.3390/biomedicines9111511 34829740 10.3390/biomedicines9111511 PMC8615182

[9] Grozdev I, Korman N, Tsankov N (2014) Psoriasis as a systemic disease. Clin Dermatol 32:343–350. 10.1016/j.clindermatol.2013.11.001 24767182 10.1016/j.clindermatol.2013.11.001

[10] Armstrong AW, Read C (2020) Pathophysiology, clinical presentation, and treatment of psoriasis: a review. JAMA 323:1945–1960. 10.1001/jama.2020.4006 32427307 10.1001/jama.2020.4006

[11] Sommer DM, Jenisch S, Suchan M et al (2007) Increased prevalence of the metabolic syndrome in patients with moderate to severe psoriasis. Arch Dermatol Res 298:321–328. 10.1007/s00403-006-0703-z 10.1007/s00403-006-0703-z 17021763

[12] Boehncke WH, Boehncke S, Tobin AM, Kirby B (2011) The “psoriatic march”: a concept of how severe psoriasis may drive cardiovascular comorbidity. Exp Dermatol 20:303–307. 10.1111/j.1600-0625.2011.01261.x 21410760 10.1111/j.1600-0625.2011.01261.x

[13] Watanabe T, Konii H, Sato K (2018) Emerging roles of cardiotrophin-1 in the pathogenesis and biomarker of atherosclerosis. J 1:94–105. 10.3390/j1010010

[14] Calabrò P, Limongelli G, Riegler L et al (2009) Novel insights into the role of cardiotrophin-1 in cardiovascular diseases. J Mol Cell Cardiol 46:142–148. 10.1016/j.yjmcc.2008.11.002 19059413 10.1016/j.yjmcc.2008.11.002

[15] Konii H, Sato K, Kikuchi S et al (2013) Stimulatory effects of cardiotrophin 1 on atherosclerosis. Hypertension 62:942–950. 10.1161/HYPERTENSIONAHA.113.01653 24041953 10.1161/HYPERTENSIONAHA.113.01653

[16] López B, González A, Querejeta R et al (2009) Association of plasma cardiotrophin-1 with stage C heart failure in hypertensive patients: potential diagnostic implications. J Hypertens 27:418–424. 10.1097/HJH.0b013e32831ac981 19155793 10.1097/HJH.0b013e32831ac981

[17] Song K, Wang S, Huang B et al (2014) Plasma cardiotrophin-1 levels are associated with hypertensive heart disease: a meta-analysis. J Clin Hypertens (Greenwich) 16:686–692. 10.1111/jch.12376 25052897 10.1111/jch.12376 PMC4159421

[18] Ashcroft DM, Li Wan Po A, Williams HC, Griffiths CEM (1999) Clinical measures of disease severity and outcome in psoriasis: a critical appraisal of their quality. Br J Dermatol 141:185–191. 10.1046/j.1365-2133.1999.02963.x 10468786 10.1046/j.1365-2133.1999.02963.x

[19] Talwar S, Squire IB, Downie PF et al (2000) Elevated circulating cardiotrophin-1 in heart failure: relationship with parameters of left ventricular systolic dysfunction. Clin Sci Lond 99:83–88 10887061

[20] López B, Castellano JM, González A et al (2007) Association of increased plasma cardiotrophin-1 with inappropriate left ventricular mass in essential hypertension. Hypertension 50:977–983. 10.1161/hypertensionaha.107.098111 17846346 10.1161/HYPERTENSIONAHA.107.098111

[21] Natal C, Fortuño MA, Restituto P et al (2008) Cardiotrophin-1 is expressed in adipose tissue and upregulated in the metabolic syndrome. Am J Physiol Endocrinol Metab 294:E52-60. 10.1152/ajpendo.00506.2007 17940213 10.1152/ajpendo.00506.2007

[22] López-Yoldi M, Moreno-Aliaga MJ, Bustos M (2015) Cardiotrophin-1: a multifaceted cytokine. Cytokine Growth Factor Rev 26:523–532. 10.1016/j.cytogfr.2015.07.009 26188636 10.1016/j.cytogfr.2015.07.009

[23] Stejskal D, Ruzicka V (2008) Cardiotrophin-1. Review. Biomed Pap Med Fac Univ Palacky Olomouc Czech Repub 152:9–19. 10.5507/bp.2008.002 18795069 10.5507/bp.2008.002

[24] Takahashi H, Tsuji H, Hashimoto Y et al (2010) Serum cytokines and growth factor levels in Japanese patients with psoriasis. Clin Exp Dermatol 35:645–649. 10.1111/j.1365-2230.2009.03704.x 19843085 10.1111/j.1365-2230.2009.03704.x

[25] Yilmaz SB, Cicek N, Coskun M et al (2012) Serum and tissue levels of IL-17 in different clinical subtypes of psoriasis. Arch Dermatol Res 304:465–469. 10.1007/s00403-012-1229-1 22426986 10.1007/s00403-012-1229-1

[26] Morsy H, Hasaballa A, Awad S (2019) Interleukin-17 levels in patients with psoriasis with or without metabolic syndrome. J Egypt Womens Dermatol Soc 16:164–169. 10.4103/jewd.Jewd_29_19

[27] Varricchi G, Granata F, Loffredo S et al (2015) Angiogenesis and lymphangiogenesis in inflammatory skin disorders. J Am Acad Dermatol 73:144–153. 10.1016/j.jaad.2015.03.041 25922287 10.1016/j.jaad.2015.03.041

[28] Krueger JG, Brunner PM (2018) Interleukin-17 alters the biology of many cell types involved in the genesis of psoriasis, systemic inflammation and associated comorbidities. Exp Dermatol 27:115–123. 10.1111/exd.13467 29152791 10.1111/exd.13467

[29] Midde HS, Priyadarssini M, Rajappa M et al (2021) Interleukin-9 serves as a key link between systemic inflammation and angiogenesis in psoriasis. Clin Exp Dermatol 46:50–57. 10.1111/ced.14335 32516443 10.1111/ced.14335

[30] Johansen C, Usher PA, Kjellerup RB et al (2009) Characterization of the interleukin-17 isoforms and receptors in lesional psoriatic skin. Br J Dermatol 160:319–324. 10.1111/j.1365-2133.2008.08902.x 19016708 10.1111/j.1365-2133.2008.08902.x

[31] Schwarz CW, Näslund-Koch C, Zachariae C et al (2025) Adverse events and immune response in psoriasis patients receiving interleukin-17 inhibitors. Acta Derm Venereol 105:adv43685. 10.2340/actadv.v105.43685 40824162 10.2340/actadv.v105.43685 PMC12376385

[32] Soeters PB, Wolfe RR, Shenkin A (2019) Hypoalbuminemia: pathogenesis and clinical significance. JPEN J Parenter Enteral Nutr 43:181–193. 10.1002/jpen.1451 30288759 10.1002/jpen.1451 PMC7379941

[33] Tan Y, Huang Z, Li H, Yao H, Fu Y, Wu X, Lin C, Lai Z, Yang G, Jing C (2024) Association between psoriasis and renal functions: an integration study of observational study and Mendelian randomization. Biomedicines. 10.3390/biomedicines12010249 38275420 10.3390/biomedicines12010249 PMC10813483

[34] Zúñiga LA, Shen WJ, Joyce-Shaikh B et al (2010) IL-17 regulates adipogenesis, glucose homeostasis, and obesity. J Immunol 185:6947–6959. 10.4049/jimmunol.1001269 21037091 10.4049/jimmunol.1001269 PMC3001125

